# COVID‐19 Vaccination and the Risk of Migraine Chronification: A Comparative Cohort Study of Vaccinated and Unvaccinated Patients With Episodic Migraine

**DOI:** 10.1002/brb3.71881

**Published:** 2026-10-30

**Authors:** Mostafa Meshref, Hossam Younis, Khabab Abbasher Hussien Mohamed Ahmed, Elsayed Abed, Abdallah Abbas, Yehia Nabil, Abdullah Ashraf Hamad, Muhammad Atallah Gad, Nour Shaheen, Mahmoud Farid Abdelmonem, Ahmed Gaballa, Ahmed Kohail, Mohamed Mahmoud Ahmed, Abdelsalam Abdullah Ali, Abdelrahman R. Youssef, Khaled Moghib

**Affiliations:** ^1^ Neurology Department, Faculty of Medicine Al‐Azhar University Cairo Egypt; ^2^ Neuroscience Department King Fahd Armed Forces Hospital Jeddah Saudi Arabia; ^3^ Faculty of Medicine University of Khartoum Khartoum Sudan; ^4^ Department of Pathophysiology, School of Medicine St. George's University St. George's Grenada, West Indies; ^5^ Faculty of Medicine Al‐Azhar University Damietta Egypt; ^6^ Faculty of Medicine Zagazig University Zagazig Egypt; ^7^ Faculty of Medicine Menoufia University Shibin El Kom Egypt; ^8^ Faculty of Medicine Alexandria University Alexandria Egypt; ^9^ Department of Biochemistry and Molecular Biology, Faculty of Medicine Al‐Azhar University Cairo Egypt; ^10^ Faculty of Medicine Minia University Minia Egypt; ^11^ Faculty of Medicine, Kasr Al‐Ainy Cairo University Cairo Egypt

**Keywords:** cohort study, COVID‐19 vaccination, episodic migraine, migraine chronification

## Abstract

**Introduction:**

Headache is among the most frequently reported adverse events after COVID‐19 vaccination, and patients with migraine often fear that vaccination will worsen their disease or accelerate the transition from episodic to chronic migraine. Controlled data comparing vaccinated and unvaccinated patients with migraine are scarce.

**Methods:**

We conducted a comparative prospective cohort study of 60 adults with pre‐existing episodic migraine, classified at enrolment as vaccinated (*n* = 30) or unvaccinated (*n* = 30) against COVID‐19, at a single neuroscience clinic in Jeddah, Saudi Arabia, and followed for 6 months. Migraine status (episodic or chronic, per ICHD‐3) was assessed at 3 and 6 months. Between‐group chronification was compared using Fisher's exact test with odds ratios (ORs) and 95% confidence intervals (CIs); patient‐level transitions were also examined. No a priori sample‐size calculation was performed.

**Results:**

There were no statistically significant baseline differences between groups in age (30.3 ± 5.07 vs. 30.5 ± 4.46 years; *p* = 0.87), sex (*p* = 0.78), or preventive medication (*p* = 0.58). Provisional chronic‐frequency status was recorded in 5/30 vaccinated versus 11/30 unvaccinated patients at 3 months (OR 0.35, 95% CI 0.10–1.16; *p* = 0.14), and chronic migraine in 3/30 versus 6/30 at 6 months (OR 0.44, 95% CI 0.10–1.97; *p* = 0.47). All such transitions occurred by 3 months, with no new cases between 3 and 6 months; the confidence intervals were wide and included no difference.

**Conclusions:**

In this small observational cohort, no statistically significant association was detected between COVID‐19 vaccination and migraine chronification. The wide confidence intervals preclude firm conclusions regarding either harm or benefit, and larger, adequately powered studies are needed.

## Graphical abstract

In a controlled cohort of 60 patients with episodic migraine, migraine chronification over 6 months did not differ significantly between 30 vaccinated and 30 unvaccinated individuals. A chronic‐frequency outcome was numerically lower after vaccination, but the confidence intervals were wide, so neither harm nor benefit can be confirmed. Where it occurred, chronification appeared early and was often transient.

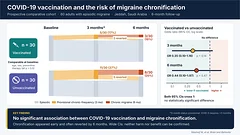

**Abbreviations**
CI: confidence interval
COVID‐19: coronavirus disease 2019
HIT‐6: Headache Impact Test‐6
ICHD‐3: International Classification of Headache Disorders, third edition
IQR: interquartile range
MIDAS: Migraine Disability Assessment
OR: odds ratio
REC: Research Ethics Committee
SARS‐CoV‐2: severe acute respiratory syndrome coronavirus 2
SD: standard deviation
STROBE: Strengthening the Reporting of Observational Studies in Epidemiology

## 1 Introduction

Coronavirus disease 2019 (COVID‐19), caused by severe acute respiratory syndrome coronavirus 2 (SARS‐CoV‐2), prompted an unprecedented global vaccination program that has substantially reduced severe disease and death (Fiolet et al. 2022; World Health Organization 2020). As with any vaccine, COVID‐19 vaccines are accompanied by mostly mild, self‐limiting adverse events, of which headache is one of the most frequently reported neurological symptoms (Castaldo et al. 2022). For the large number of people who live with migraine, this raises an understandable concern: might vaccination trigger, intensify, or prolong their attacks, or even hasten the transition from episodic to chronic disease?

Migraine is a common and disabling primary headache disorder. The International Classification of Headache Disorders, third edition (ICHD‐3), distinguishes episodic migraine from chronic migraine, the latter defined as headache on at least 15 days per month for more than 3 months, with migrainous features on at least eight of those days (Headache Classification Committee of the International Headache Society 2018). Progression from episodic to chronic migraine—chronification—matters clinically, being associated with greater disability, higher healthcare use, and poorer quality of life. Recognized drivers include older age, female sex, high baseline attack frequency, medication overuse, obesity, depression, and stressful life events (May and Schulte 2016).

Whether COVID‐19 vaccination contributes to migraine worsening remains unsettled. Questionnaire‐based studies have reported that some patients experience more intense or more frequent headaches after vaccination (Sekiguchi et al. 2022; Silvestro et al. 2021), whereas longitudinal data suggest that any change in monthly migraine days is often small or transient (van der Arend et al. 2023). Others have argued that deterioration attributed to infection or vaccination may partly reflect a nocebo response and the broader stress of the pandemic rather than a direct biological effect (Melgarejo et al. 2023). A recurring limitation across this literature is the absence of a concurrent unvaccinated comparison group: without one, it is difficult to separate any vaccine‐related change from the background rate at which episodic migraine becomes chronic over the same interval.

To address this gap, we compared the clinical course of two concurrent cohorts of patients with pre‐existing episodic migraine—one vaccinated against COVID‐19 and one unvaccinated—over 6 months. Our primary aim was to determine whether the incidence of migraine chronification at 3 and 6 months differed between vaccinated and unvaccinated patients; secondarily, we examined baseline characteristics associated with chronification within each cohort.

## 2 Methods

### 2.1 Study Design and Setting

We conducted a comparative, prospective observational cohort study at the Neuroscience Department of King Fahd Armed Forces Hospital, Jeddah, Saudi Arabia. Eligible adults with episodic migraine attending the department's headache clinic were enrolled following Research Ethics Committee approval in June 2022. The exact recruitment window, and confirmation that enrolment was consecutive, could not be verified from the analytic dataset available for this study and are noted as a reporting limitation. The study is reported in accordance with the Strengthening the Reporting of Observational Studies in Epidemiology (STROBE) guidance for cohort studies (von Elm et al. 2008).

### 2.2 Participants and Study Groups

Sixty adults with an established diagnosis of episodic migraine were enrolled and classified into two cohorts according to COVID‐19 vaccination status at enrolment. Patients who had received at least one dose of a COVID‐19 vaccine were assigned to the vaccinated cohort (*n* = 30), and those who had received no dose at enrolment to the unvaccinated cohort (*n* = 30). The cohorts were therefore defined by baseline vaccination status; vaccination status was not systematically re‐ascertained at each subsequent visit, so crossover of exposure during follow‐up cannot be excluded and is addressed in the Limitations. Both cohorts were recruited and followed over the same calendar period and assessed at the same intervals. At enrolment we recorded age, sex, migraine type, and preventive migraine medication. Detailed vaccine characteristics (product, number of doses, and dates) and the interval between vaccination and baseline assessment were not recorded in a structured manner, and prior or incident SARS‐CoV‐2 infection during follow‐up was not systematically ascertained; these variables were therefore unavailable for analysis and are addressed in the Limitations.

### 2.3 Eligibility Criteria

Patients were eligible if they were adults with a documented diagnosis of episodic migraine before enrolment and had complete follow‐up data at both 3 and 6 months. We excluded patients with chronic migraine antedating enrolment, another primary or secondary headache disorder, a major neurological or medical condition likely to confound headache classification, or incomplete follow‐up.

### 2.4 Outcome Definition and Assessment

Migraine was classified according to ICHD‐3 (Headache Classification Committee of the International Headache Society 2018). Episodic migraine was defined as headache on fewer than 15 days per month; chronic migraine as headache on 15 or more days per month for more than 3 months, with migrainous features on at least 8 days per month. Migraine status was recorded at each scheduled follow‐up assessment using this framework. The analytic dataset did not include a structured variable documenting the exact method used to ascertain monthly headache and migraine‐day frequency (for example, a prospective headache diary versus recall or clinical record) or the identity of the clinician who assigned each classification; this ascertainment limitation is acknowledged below.

An important methodological point concerns the 3‐month assessment. Because the ICHD‐3 temporal criterion for chronic migraine requires the qualifying headache pattern to persist for more than 3 months, the classification made at the 3‐month visit represents a provisional chronic‐frequency category based on the monthly headache frequency recorded at that time point; the full ICHD‐3 temporal criterion could be satisfied only at the 6‐month assessment. This distinction is carried through the interpretation of our findings and is acknowledged as a limitation.

### 2.5 Variables and Outcomes

The primary outcome was migraine status (episodic or chronic) at 3 and 6 months, compared between the vaccinated and unvaccinated cohorts. Baseline covariates were age, sex, and preventive medication (amitriptyline, propranolol, topiramate, or a combination). In secondary analyses we examined the association between these baseline characteristics and migraine status within each cohort. Several clinically relevant variables were not captured in the available dataset—baseline monthly headache‐day and migraine‐day frequency, attack severity, validated disability scores (for example, MIDAS or HIT‐6), changes in preventive or acute migraine treatment during follow‐up, and acute‐medication overuse—and these are addressed in the Limitations.

### 2.6 Statistical Analysis

Continuous variables are summarized as mean ± standard deviation (SD) and as median with interquartile range (IQR); categorical variables as counts and percentages. The distribution of age was examined for normality using the Shapiro–Wilk test; because age was normally distributed in both cohorts (*p* = 0.15 and *p* = 0.61), comparisons of age between groups and between status categories used the independent‐samples *t*‐test. Categorical comparisons, including chronification between cohorts and associations with sex and medication, used Fisher's exact test; for the primary between‐cohort comparison we report odds ratios with 95% confidence intervals and the absolute risk difference, which we use as the principal representation of statistical uncertainty. To account for the repeated assessment of the same participants, we examined individual patient‐level transitions in migraine status across baseline, 3 months, and 6 months, and compared the change in status between 3 and 6 months within each cohort using the exact McNemar test; a formal mixed‐effects model was not fitted because events were sparse and no new events occurred after the 3‐month assessment. No a priori sample‐size calculation was performed, and the absence of a statistically significant association does not establish equivalence, non‐inferiority, or a protective effect. All tests were two‐sided, with *p* < 0.05 considered statistically significant. Analyses were performed in Python 3.12 using the SciPy, pandas, and statsmodels libraries.

### 2.7 Ethical Considerations

The study was approved by the Research Ethics Committee of Armed Forces Hospitals, Jeddah, Saudi Arabia, on June 22, 2022 (application 2022–35; reference REC 517), and was conducted in accordance with the ethical principles of the Declaration of Helsinki (World Medical Association 2013). Written informed consent was obtained from all participants before enrolment.

## 3 Results

### 3.1 Baseline Characteristics

Sixty patients with pre‐existing episodic migraine were enrolled, 30 in each cohort, and all had episodic migraine at baseline (Figure 1; participant flow shown in Figure 2). There were no statistically significant baseline differences between the vaccinated and unvaccinated cohorts in the measured variables. Mean age was 30.3 ± 5.07 years in the vaccinated group and 30.5 ± 4.46 years in the unvaccinated group (*p* = 0.87), with identical median ages of 30 years. Women comprised 21 of 30 (70.0%) of the vaccinated and 19 of 30 (63.3%) of the unvaccinated cohort (*p* = 0.78), and the distribution of preventive medication was similar between groups (*p* = 0.58) (Table 1). Baseline monthly headache‐day and migraine‐day frequencies were not available in the dataset, so comparability of baseline headache burden between the cohorts could not be established. Because the cohorts were not formally matched and important confounders were not measured, residual confounding cannot be excluded.

**FIGURE 1:**
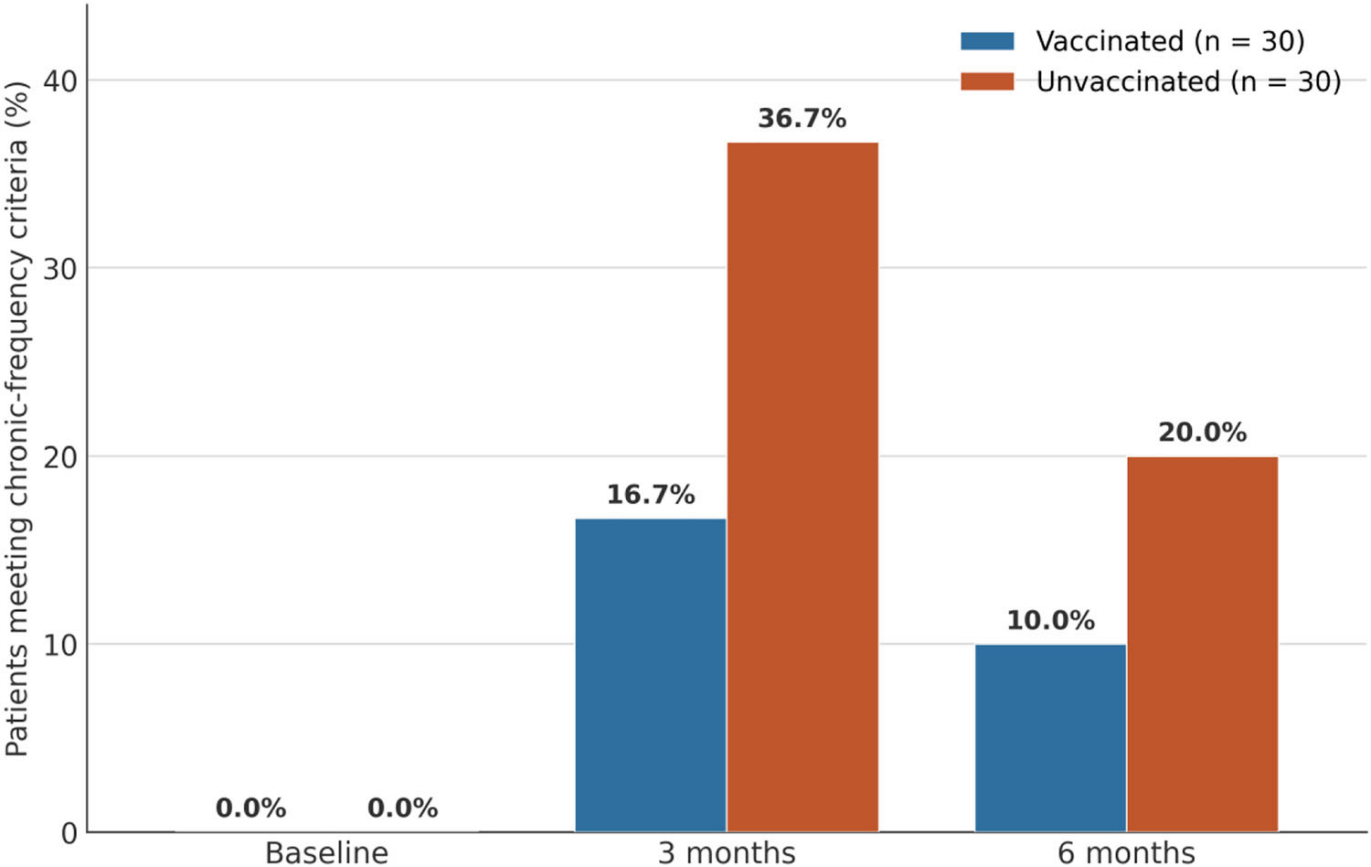
Proportion of patients meeting chronic‐frequency criteria at baseline and at 3 and 6 months in the vaccinated (*n* = 30) and unvaccinated (*n* = 30) cohorts. At 3 months, the value reflects provisional chronic‐frequency status, since the ICHD‐3 duration criterion could be met only at 6 months (chronic migraine). Both cohorts were entirely episodic at baseline.

**FIGURE 2:**
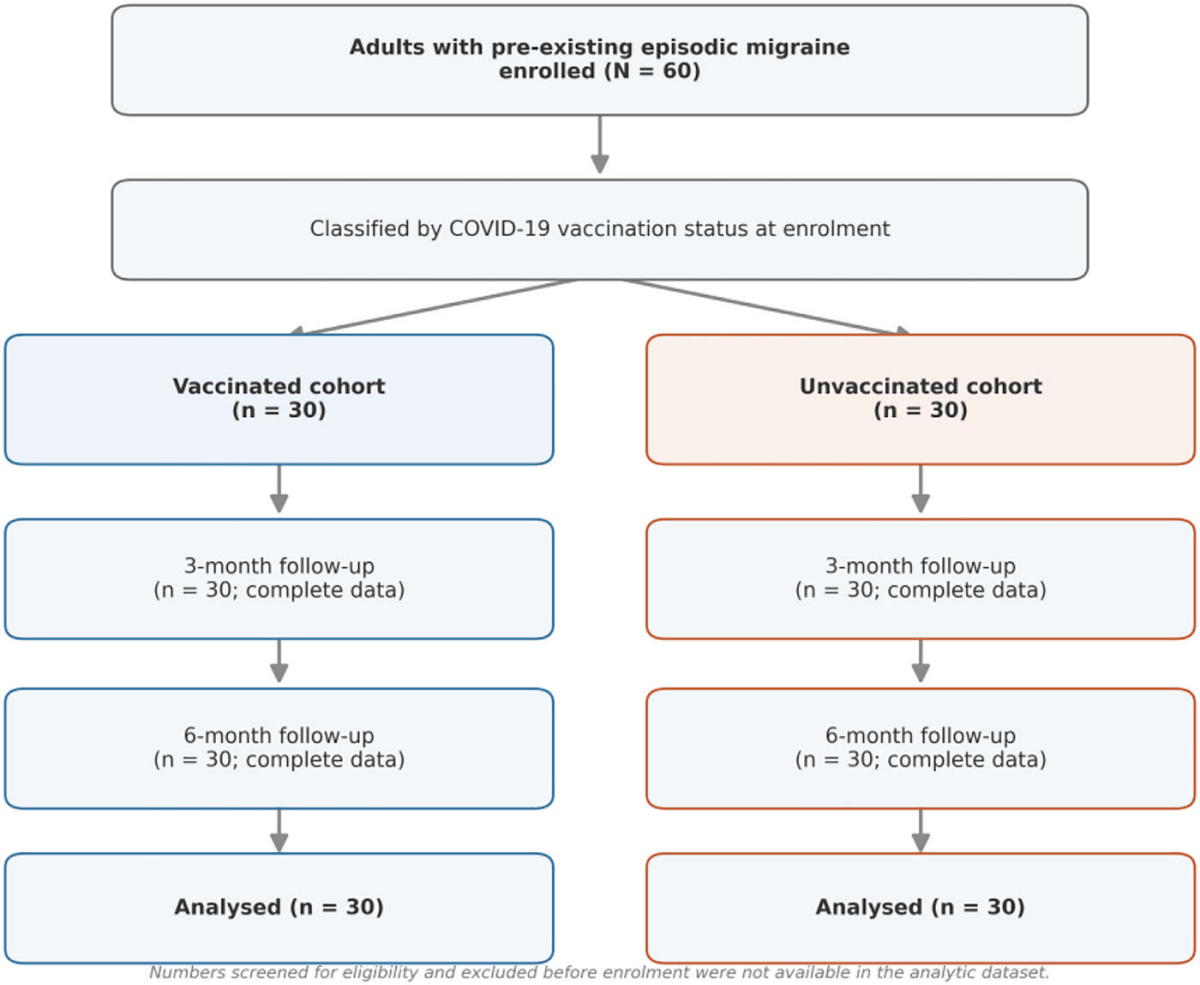
Participant flow through the study (STROBE), showing enrolment, classification by COVID‐19 vaccination status at enrolment, follow‐up at 3 and 6 months, and the numbers analyzed. The numbers screened for eligibility and excluded before enrolment were not available in the analytic dataset; the diagram therefore begins at enrolment.

**TABLE 1:** Baseline characteristics of the vaccinated and unvaccinated cohorts.

Characteristic	Vaccinated (*n* = 30)	Unvaccinated (*n* = 30)	*p* value
Age, years, mean ± SD	30.3 ± 5.07	30.5 ± 4.46	0.87
Age, years, median (IQR)	30 (28–35)	30 (28–34)	—
Female, *n* (%)	21 (70.0)	19 (63.3)	0.78
Male, *n* (%)	9 (30.0)	11 (36.7)	—
Amitriptyline, *n* (%)	11 (36.7)	13 (43.3)	0.58
Propranolol, *n* (%)	8 (26.7)	8 (26.7)	—
Topiramate, *n* (%)	9 (30.0)	5 (16.7)	—
Combination therapy, *n* (%)	2 (6.7)	4 (13.3)	—
Episodic migraine at baseline, *n* (%)	30 (100.0)	30 (100.0)	—

*Note*: Age compared with the independent‐samples *t*‐test (age was normally distributed by the Shapiro–Wilk test in both cohorts); sex and preventive medication distribution compared with Fisher's exact test. No statistically significant baseline differences were observed in the measured variables. Baseline monthly headache‐day and migraine‐day frequencies were not available in the dataset, so comparability of baseline headache burden could not be established. IQR, interquartile range; SD, standard deviation.

### 3.2 Provisional Chronic‐Frequency Status at 3 Months

At 3 months, provisional chronic‐frequency status was recorded in 5 of 30 vaccinated patients (16.7%) and 11 of 30 unvaccinated patients (36.7%). The between‐cohort difference was not statistically significant (OR 0.35, 95% CI 0.10–1.16; absolute risk difference −20.0%, 95% CI −41.8% to +1.8%; *p* = 0.14) (Table 2; Figure 1). The confidence intervals were wide and included no difference.

**TABLE 2:** Provisional chronic‐frequency status at 3 months and chronic migraine at 6 months in the vaccinated versus unvaccinated cohorts.

Outcome and time point	Vaccinated, *n* (%)	Unvaccinated, *n* (%)	OR (95% CI)	*p* value
Provisional chronic‐frequency status, 3 months	5 (16.7)	11 (36.7)	0.35 (0.10–1.16)	0.14
Chronic migraine, 6 months	3 (10.0)	6 (20.0)	0.44 (0.10–1.97)	0.47

*Note*: Outcomes compared between cohorts using Fisher's exact test. Because the ICHD‐3 duration criterion for chronic migraine requires the pattern to persist for more than 3 months, the 3‐month outcome is reported as provisional chronic‐frequency status, and only the 6‐month outcome represents chronic migraine. Odds ratios (vaccinated vs. unvaccinated) with 95% confidence intervals; the absolute risk difference was −20.0% (95% CI −41.8% to +1.8%) at 3 months and −10.0% (95% CI −27.9% to +7.9%) at 6 months. Both intervals include the null, and the non‐significant results do not establish equivalence or a protective effect. All patients were episodic at baseline. CI, confidence interval; OR, odds ratio.

### 3.3 Migraine Chronification at 6 Months

At 6 months, chronic migraine was present in 3 of 30 vaccinated patients (10.0%) and 6 of 30 unvaccinated patients (20.0%). This difference was again not statistically significant (OR 0.44, 95% CI 0.10–1.97; absolute risk difference −10.0%, 95% CI −27.9% to +7.9%; *p* = 0.47) (Table 2; Figure 1); here too the confidence intervals were wide and included no difference.

### 3.4 Patient‐Level Transitions Between 3 and 6 Months

Examination of individual trajectories showed that all transitions occurred by the 3‐month assessment: no participant moved from episodic to chronic‐frequency status between 3 and 6 months in either cohort. Reversion to episodic status occurred in both groups—2 of the 5 vaccinated and 5 of the 11 unvaccinated patients who met provisional chronic‐frequency status at 3 months had returned to episodic status by 6 months. The exact McNemar test for change in status between 3 and 6 months was not statistically significant in either cohort (vaccinated *p* = 0.50; unvaccinated *p* = 0.06) (Table 3).

**TABLE 3:** Patient‐level transitions in migraine status across baseline, 3 months, and 6 months.

Trajectory (baseline → 3 months → 6 months)	Vaccinated, *n* (%)	Unvaccinated, *n* (%)
Episodic at all three time points	25 (83.3)	19 (63.3)
Provisional chronic‐frequency status at 3 months, chronic migraine at 6 months	3 (10.0)	6 (20.0)
Provisional chronic‐frequency status at 3 months, episodic at 6 months	2 (6.7)	5 (16.7)
Episodic at 3 months, chronic migraine at 6 months	0 (0.0)	0 (0.0)

*Note*: All chronic‐frequency outcomes appeared by the 3‐month assessment; no participant moved from episodic to chronic‐frequency status between 3 and 6 months. The 3‐month outcome is provisional chronic‐frequency status, since the ICHD‐3 duration criterion could be met only at 6 months (chronic migraine). Reversion to episodic status occurred in both cohorts. Exact McNemar test for change in status between 3 and 6 months: vaccinated *p* = 0.50; unvaccinated *p* = 0.06.

### 3.5 Baseline Factors and Migraine Status Within Cohorts

Within the vaccinated cohort, the three patients who were chronic at 6 months were older than those who remained episodic (36.3 ± 2.08 vs. 29.6 ± 4.87 years; *p* = 0.03); a similar, non‐significant direction was seen at 3 months (33.8 ± 5.67 vs. 29.6 ± 4.76 years; *p* = 0.09). In the unvaccinated cohort, chronic patients were also somewhat older, without reaching significance at either time point. Because this comparison rests on only three chronic cases in the vaccinated cohort, it is reported here as an exploratory observation and is not presented as a separate table, so as not to over‐emphasize a finding based on so few events. Sex and preventive medication use were not associated with migraine status at 6 months in either cohort (all *p* > 0.05).

## 4 Discussion

In this comparative cohort of 60 patients with pre‐existing episodic migraine, no statistically significant association was detected between COVID‐19 vaccination and migraine chronification at 3 or 6 months. A chronic‐frequency outcome—provisional at 3 months and meeting full ICHD‐3 criteria for chronic migraine at 6 months—was numerically less frequent in vaccinated than unvaccinated patients at both time points, but the confidence intervals were wide and crossed the null, so the estimates are imprecise. These data therefore indicate that an increased risk of chronification after vaccination was not observed; they do not establish equivalence, non‐inferiority, or a protective effect of vaccination.

The principal methodological contribution of this study is the inclusion of a concurrent unvaccinated comparison group, which much of the earlier literature lacks. Episodic migraine fluctuates over time, and a proportion of patients will meet chronic criteria over any given interval regardless of vaccination; indeed, more than a third of our unvaccinated patients met the 3‐month threshold. Single‐arm post‐vaccination series cannot separate vaccine‐related change from this background fluctuation, which risks misattributing ordinary variation to the vaccine. Our data suggest that the chronification observed after vaccination did not exceed that seen without it, although the limited precision of our estimates must be kept in view.

These observations are broadly compatible with the wider literature. Headache is a recognized adverse event after SARS‐CoV‐2 vaccination (Castaldo et al. 2022), and questionnaire studies by Silvestro et al. (2021) and Sekiguchi et al. (2022) found that some patients with migraine or a prior headache history report post‐vaccination worsening. Longitudinal data from van der Arend et al. (2023) indicate that COVID‐19 vaccination does not produce a durable increase in monthly migraine days, and Melgarejo et al. (2023) proposed that much of the deterioration reported after infection or vaccination reflects a nocebo effect amplified by pandemic‐related stress. Our controlled comparison is consistent with this literature and adds the observation that unvaccinated patients did not fare better than vaccinated ones over the same interval.

The pattern of transitions is informative. All chronic‐frequency outcomes appeared by 3 months, no participant newly met these criteria between 3 and 6 months, and a substantial proportion of those meeting provisional chronic‐frequency status at 3 months had reverted to episodic status by 6 months in both cohorts. This is more consistent with transient fluctuation in headache frequency than with sustained, progressive chronification, and it reinforces the importance of the ICHD‐3 temporal criterion, which the 3‐month classification could not fully satisfy.

Within the vaccinated cohort, older age was statistically associated with chronic status at 6 months. Although age is an established contributor to chronification (May and Schulte 2016), this finding rests on only three chronic cases, the estimate is unstable, and it should be regarded as hypothesis‐generating rather than as a reliable predictor. A similar, non‐significant age gradient in the unvaccinated cohort suggests that any age effect reflects migraine chronification in general rather than anything specific to vaccination.

Any short‐term post‐vaccination headache is biologically explicable through transient innate and adaptive immune activation and cytokine release acting on trigeminovascular pain pathways in predisposed individuals (Teijaro and Farber 2021). A comprehensive review of the neurological manifestations of COVID‐19 and its vaccines concluded that post‐vaccination neurological symptoms are usually mild and transient, with serious complications rare (Ahmed et al. 2025)—a picture consistent with a self‐limiting effect rather than durable disease modification.

Clinically, these findings indicate that no statistically significant increase in the short‐term risk of migraine chronification was detected relative to remaining unvaccinated. Because the estimates were imprecise, however, neither harm nor benefit can be firmly excluded, and the results should not be presented to patients as definitive reassurance. Patients who report sustained worsening after vaccination still merit careful evaluation, optimization of preventive treatment, and screening for medication overuse, in keeping with standard migraine care.

### 4.1 Strengths

The principal strength of this study is the concurrent unvaccinated comparison group, which allows any vaccine‐associated change to be judged against the background rate of chronification. Follow‐up was prospective and applied predefined ICHD‐3 criteria at two clinically meaningful time points, all 60 participants included in the analytic cohort had complete data at 3 and 6 months, and the cohorts showed no significant baseline differences in the measured variables.

### 4.2 Limitations

Several limitations temper these findings. First, the sample was small (30 per cohort) with few events and no a priori sample‐size calculation; consequently, the estimates were imprecise and the wide confidence intervals cannot distinguish a true absence of effect from a clinically relevant one. Second, group assignment was not randomized and the cohorts were defined by baseline vaccination status; because vaccination status was not re‐ascertained at each visit, crossover of exposure during follow‐up cannot be excluded, and unmeasured confounders—including baseline monthly migraine and headache‐day frequency, attack severity, validated disability scores, acute‐medication use and medication overuse, prior or incident SARS‐CoV‐2 infection, and the reasons for accepting or declining vaccination—could have influenced the results. Third, vaccine characteristics (type, number of doses, timing, and the interval to symptom change) were not captured. Fourth, the 3‐month classification of chronic migraine was provisional, because the ICHD‐3 temporal criterion can be met only after more than 3 months; the full criterion was applied at 6 months. Fifth, the dataset did not document the exact method used to ascertain headache frequency or the clinician who assigned each classification, and changes in preventive or acute treatment during follow‐up were not recorded. Sixth, the exact recruitment window, confirmation of consecutive enrolment, and the numbers screened and excluded before enrolment were not available, so the participant‐flow diagram begins at enrolment. Finally, the study was conducted at a single center, migraine status was analyzed as a category rather than as a continuous measure of headache burden, and follow‐up ended at 6 months.

### 4.3 Future Directions

Larger, adequately powered, and ideally multicenter studies with randomized or carefully matched designs are needed to determine whether COVID‐19 vaccination has any effect on migraine chronification. Future work should record vaccine‐specific details, prior and incident infection, baseline and follow‐up monthly migraine days from prospective diaries, attack severity and duration, changes in preventive and acute therapy, and validated disability measures, and should apply longitudinal modelling and, where appropriate, a formal equivalence or non‐inferiority framework.

## 5 Conclusions

In this small observational cohort, no statistically significant association was detected between COVID‐19 vaccination and migraine chronification in patients with pre‐existing episodic migraine. The wide confidence intervals preclude firm conclusions regarding either harm or benefit. Where chronification occurred, it appeared early and was frequently transient, and the association between older age and chronic status within the vaccinated cohort was an exploratory observation only. Larger, adequately powered, and ideally longitudinal studies are needed to establish whether COVID‐19 vaccination has any effect on migraine chronification.

## Author Contributions

Contributions are reported using the CRediT (Contributor Roles Taxonomy) framework, with author initials as follows: **Mostafa Meshref** (M.M.), **Hossam Younis** (H.Y.), **Khabab Abbasher Hussien Mohamed Ahmed** (K.A.H.M.A.), **Elsayed Abed** (E.A.), **Abdallah Abbas** (A.A.), **Yehia Nabil** (Y.N.), **Abdullah Ashraf Hamad** (A.A.H.), **Muhammad Atallah Gad** (M.A.G.), **Nour Shaheen** (N.S.), **Mahmoud Farid Abdelmonem** (M.F.A.), **Ahmed Gaballa** (A.G.), **Ahmed Kohail** (A.K.), **Mohamed Mahmoud Ahmed** (M.M.A.), Abdelsalam Abdullah Ali (A.A.A.), **Abdelrahman R. Youssef** (A.R.Y.), and **Khaled Moghib** (K.M.). **Conceptualization**: M.M. and H.Y. **Methodology**: M.M., H.Y., E.A., and M.A.G. **Investigation and clinical data collection**: H.Y. and M.M. **Data curation**: A.A., Y.N., A.A.H., and N.S. **Formal analysis**: A.A., M.F.A., and A.R.Y. **Visualization**: A.G., A.K., M.M.A., and A.A.A. **Validation**: E.A., M.A.G., and K.M. **Writing – original draft**: K.A.H.M.A., A.A., Y.N., A.A.H., and N.S. **Writing – review and editing**: M.M., H.Y., K.A.H.M.A., E.A., M.F.A., A.G., A.K., M.M.A., A.A.A., A.R.Y., and K.M. **Supervision**: M.M. and H.Y. **Project administration**: K.A.H.M.A. and M.M. **Resources**: H.Y. All authors had full access to the study data, read and approved the final version of the manuscript, and agree to be accountable for all aspects of the work.

## Funding

The authors have nothing to report.

## Ethics Statement

Ethical approval was granted by the Research Ethics Committee of Armed Forces Hospitals, Jeddah, Saudi Arabia, on June 22, 2022 (application 2022–35; reference REC 517). Written informed consent was obtained from all participants before inclusion.

## Conflicts of Interest

The authors declare no conflicts of interest.

## Data Availability

The data that support the findings of this study are available from the corresponding author upon reasonable request.
